# TPpred-LE: therapeutic peptide function prediction based on label embedding

**DOI:** 10.1186/s12915-023-01740-w

**Published:** 2023-10-31

**Authors:** Hongwu Lv, Ke Yan, Bin Liu

**Affiliations:** 1 School of Computer Science and Technology, Beijing Institute of Technology, Beijing, 100081 China; 2 Advanced Research Institute of Multidisciplinary Science, Beijing Institute of Technology, No. 5, South Zhongguancun Street, Haidian District, Beijing, 100081 China

**Keywords:** Therapeutic peptide prediction, Multi-label classification, Relationship information, Multi-label classifier retrain

## Abstract

**Background:**

Therapeutic peptides play an essential role in human physiology, treatment paradigms and bio-pharmacy. Several computational methods have been developed to identify the functions of therapeutic peptides based on binary classification and multi-label classification. However, these methods fail to explicitly exploit the relationship information among different functions, preventing the further improvement of the prediction performance. Besides, with the development of peptide detection technology, peptide functions will be more comprehensively discovered. Therefore, it is necessary to explore computational methods for detecting therapeutic peptide functions with limited labeled data.

**Results:**

In this study, a novel method called TPpred-LE based on Transformer framework was proposed for predicting therapeutic peptide multiple functions, which can explicitly extract the function correlation information by using label embedding methodology and exploit the specificity information based on function-specific classifiers. Besides, we incorporated the multi-label classifier retraining approach (MCRT) into TPpred-LE to detect the new therapeutic functions with limited labeled data. Experimental results demonstrate that TPpred-LE outperforms the other state-of-the-art methods, and TPpred-LE with MCRT is robust for the limited labeled data.

**Conclusions:**

In summary, TPpred-LE is a function-specific classifier for accurate therapeutic peptide function prediction, demonstrating the importance of the relationship information for therapeutic peptide function prediction. MCRT is a simple but effective strategy to detect functions with limited labeled data.

**Supplementary Information:**

The online version contains supplementary material available at 10.1186/s12915-023-01740-w.

## Background

Therapeutic peptides play an essential role in human physiology, treatment paradigms, and bio-pharmacy [1–3]. Over the last few decades, peptide-based therapeutics have received a great deal of attention from researchers due to their advantages in drug discovery and design [4, 5]. During the epidemic of COVID-19, therapeutic peptides have shown their potential as the agents against SARS-CoV-2 [6–8]. In addition to the anti-viral function, therapeutic peptides also show different functions, such as anti-microbial, anti-cancer, anti-inflammatory, etc. [9, 10]. The recognition of the functions of therapeutic peptides is important.

The data-driven computational methods have been widely used in therapeutic peptide function prediction over the past decade. Those methods can be categorized into two groups in terms of the methodologies: (i) binary classification methods and (ii) multi-label classification methods.

The binary classification methods usually utilize conventional machine learning predictors by employing different feature extraction methods. PEPred-Suite [9] is an efficient approach based on random forest (RF) for therapeutic peptide function prediction by integrating distinct feature descriptors for different peptide functions. PPTPP [11] is also a RF-based method, where a feature extraction method MRMD2.0 was adopted to produce and rank physicochemical property-related features. TPpred-ATMV [10] adopted multi-view learning, which assumed that different property features were derived from the common latent subspaces, and utilized the high correlation among different features to predict the peptide functions. The aforementioned methods independently constructed the specific predictor for each therapeutic peptide function, ignoring the correlation among different peptide functions.

The multi-label classification methods have attracted more and more attentions in recent years. MLBP [12] treated the prediction of bioactivate peptides as a multi-label classification task and adopted convolutional neural network (CNN) and bidirectional gated recurrent units (BiGRU) to predict the multi-functional bioactivate peptides. PrMFTP [13] introduced the attention mechanism [14] based on MLBP. However, these predictors only consider the sequence information, failing to explicitly incorporate the relationship information among multi-functional peptides, such as the correlation information and the specificity information.

As discussed above, the existing methods are suffering from two major disadvantages: (i) the existing methods failed to explicitly and accurately capture the relationship among different therapeutic peptide functions. For example, the methods based on binary classifiers only consider the specificity information of mono-functional therapeutic peptides ignoring their correlation information among different functions. (ii) For the newly sequenced therapeutic peptides, the existing computational predictors cannot accurately detect their comprehensive functions. Therefore, it is desired to recognize their unknown functions with limited labeled data.

In this study, we proposed a computational predictor called TPpred-LE for multi-functional therapeutic peptide prediction. TPpred-LE exploits the relationship among different functions based on label embedding and function-specific classifiers, including the correlation information and the specificity information. Furthermore, we proposed a multi-label classifier retraining approach (MCRT) based on the classifier retraining approach (cRT) [15], which was incorporated into TPpred-LE to detect new functions with limited labeled data. Experimental results demonstrate that TPpred-LE achieves the state-of-the-art performance.

## Results

### An overview of TPpred-LE

In this study, we exploit the prediction ability of TPpred-LE on the benchmark dataset with 15 different therapeutic peptide functions, including AMP (anti-microbial peptide), TXP (toxic peptide), ABP (anti-bacterial peptide), AIP (anti-inflammatory peptide), AVP (anti-viral peptide), ACP (anti-cancer peptide), AFP (anti-fungal peptide), DDV (drug delivery vehicle peptide), CPP (cell-penetrating peptide), CCC (cell–cell communication peptide), APP (anti-parasitic peptide), AAP (anti-angiogenic peptide), AHTP (anti-hypertensive peptide), PBP (polystyrene surface-binding peptide), and QSP (quorum sensing peptide). The benchmark dataset is divided into training dataset, validation dataset, and independent test dataset.

**Fig. 1:**
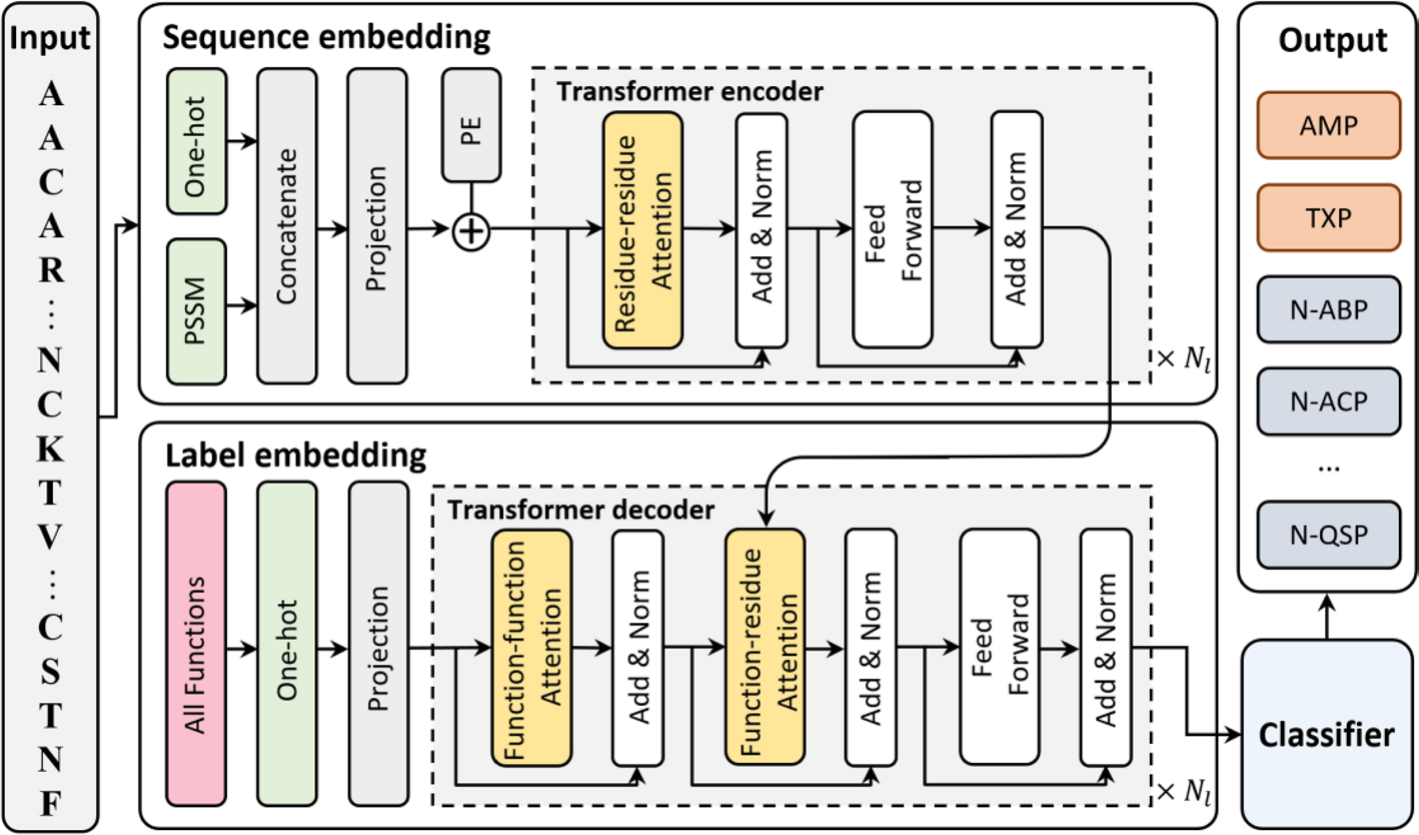
The framework of TPpred-LE

The framework of TPpred-LE is illustrated in Fig. 1. TPpred-LE contains three modules: (i) sequence embedding module, (ii) label embedding module, and (iii) classifier module. The sequence embedding module is mainly composed of the Transformer encoder, in which the residue-residue attention embeds the information relationship between any two different residues along the sequence. The Transformer decoder plays an essential role in the label embedding module, in which the function-function attention learns the relationship information between different therapeutic peptide functions, and the function-residue attention integrates the residue embedding and the function embedding. A representation vector corresponding to each function is constructed after the two embedding processes. Finally, each function is accurately predicted by the classifier module based on the representation vectors.

We use the multi-label metrics ACC_*example*_ (example-based Accuracy) and F1_*label*_ (label-based F1-score) to evaluate the overall performance of TPpred-LE [16]. Besides, we also utilize the binary classification metrics to evaluate the performance for each therapeutic peptide function prediction task in a one-versus-all form, including the area under the ROC curve (AUC) [17], Matthews’s correlation coefficient (MCC) [18], the F1 measure [19], and the K-category correlation coefficient (RkCC) [20].

### Relationship information among therapeutic peptide functions can improve the performance

We conduct ablation experiments to investigate the importance of the relationship information among different therapeutic peptide functions, including the correlation information and the specificity information. The corresponding results are listed in Table 1, from which we can see that TPpred-LE achieves the best performance, because it takes advantage of both the correlation information and the specificity information, demonstrating the importance of relationship information among therapeutic peptide functions for therapeutic peptide prediction. Specifically, TPpred-LE outperforms model E, which removes the function-specific classifiers and all functions share the single classifier, indicating that it is useful to learn a unique decision boundary by the function-specific classifiers for each function with unique feature distribution.

**Table 1:** Impact of the correlation and specificity modules on the performance of TPpred-LE evaluated on the independent dataset

Model	Correlation		Specificity	Performance	
	Label embedding	Single classifier	Function-specific classifiers	ACC_example_	F1_label_
TPpred-LE	✓	✓	✓	**0.536**	**0.422**
A^a^	*x*	✓	✓	0.510	0.391
B	✓	*x*	✓	0.503	0.392
C	*x*	*x*	✓	0.499	0.400
D	*x*	✓	*x*	0.456	0.311
E	✓	✓	*x*	0.509	0.400

^a^Integrate the two types of classifiers by the two-phase training

**Fig. 2:**
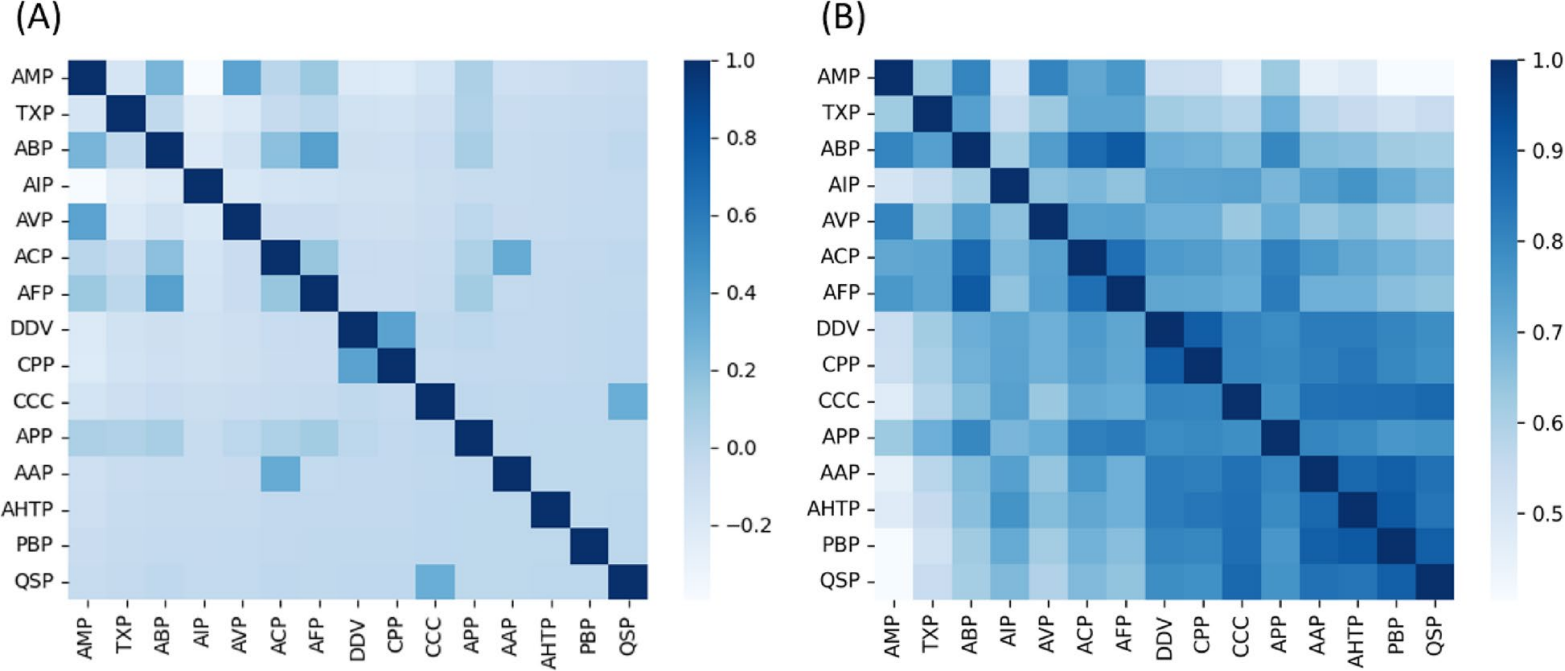
**A** Pearson’s correlation coefficient of the functions computed by the samples in the training set. **B** The average Pearson’s correlation coefficient of the function representations learned by TPpred-LE. Each element represents the correlation coefficient of the two corresponding functions. The darker elements indicate stronger relevance

We further visualize Pearson’s correlation coefficient [21] of the functions in the training set and the average Pearson’s correlation coefficient by averaging the coefficient scores of the function representations learned by the label embedding module in TPpred-LE. The detailed mathematical formulas are described in Additional file 1: Supplementary Material S1 [21]. The results are shown in Fig. 2, from which we can see the that the relevant functions tend to show similar representations, indicating that the function representations are able to capture the characteristics of the therapeutic peptide functions.

### Performance comparison among different predictors for therapeutic peptide function prediction

Most of the existing methods only predict some specific therapeutic peptide functions and treat this task as binary classification problem. In contrast, TPpred-LE is the only method for comprehensively predicting 15 different therapeutic peptide functions. The performance of TPpred-LE is measured by binary classification metrics and compared with the state-of-the-art binary classification methods for therapeutic peptide prediction, including PEPred-Suite [9], PPTPP [11], and TPpred-ATMV [10]. The results are listed in Table 2, from which we can see that TPpred-LE achieves the best performance. Because the binary classifier methods are suffering from the high false-positive rate problem (see Additional file 3: Table S1), they tend to predict the negatives as the positives. Different from these methods, TPpred-LE is simultaneously trained with 15 different therapeutic peptide functions and explicitly explores the correlation information of different therapeutic peptide functions to learn more discriminative features. As a result, TPpred-LE are obviously better than the other existing predictors, especially for predicting multi-functional therapeutic peptides with imbalanced training data. The comprehensive performance of other functions and the results are available in Additional file 3: Table S2 in terms of the Rkcc metric.

**Table 2:** The performance of various methods for predicting eight therapeutic peptide functions on the independent dataset

Function	Method	AUC	MCC	F1
AAP	PEPred-Suite^a^	0.577	0.02	0.03
	PPTPP^ab^	0.604	0.037	0.033
	TPpred-ATMV^a^	0.583	0.009	0.027
	TPpred-LE	**0.745**	**0.278**	**0.285**
ABP	PEPred-Suite^a^	0.744	0.261	0.367
	PPTPP^ab^	0.732	0.261	0.365
	TPpred-ATMV^a^	0.731	0.256	0.36
	TPpred-LE	**0.834**	**0.337**	**0.426**
ACP	PEPred-Suite^a^	0.56	0.03	0.155
	PPTPP^ab^	0.625	0.049	0.162
	TPpred-ATMV^a^	0.662	0.096	0.183
	TPpred-LE	**0.773**	**0.328**	**0.371**
AIP	PEPred-Suite^a^	0.363	− 0.19	0.18
	PPTPP^ab^	0.386	− 0.06	0.168
	TPpred-ATMV^a^	0.369	− 0.25	0.196
	TPpred-LE	**0.895**	**0.527**	**0.594**
AVP	PEPred-Suite^a^	0.382	− 0.129	0.147
	PPTPP^ab^	0.404	− 0.11	0.169
	TPpred-ATMV^a^	0.394	− 0.118	0.135
	TPpred-LE	**0.835**	**0.457**	**0.529**
CPP	PEPred-Suite^a^	0.813	0.152	0.142
	PPTPP^ab^	0.814	0.14	0.139
	TPpred-ATMV^a^	0.815	0.152	0.139
	TPpred-LE	**0.899**	**0.477**	**0.502**
PBP	PEPred-Suite^a^	0.907	0.153	0.069
	PPTPP^ab^	0.829	0.119	0.07
	TPpred-ATMV^a^	0.836	0.153	0.086
	TPpred-LE	**0.934**	**0.443**	**0.430**
QSP	PEPred-Suite^a^	0.835	0.113	0.043
	PPTPP^ab^	0.815	0.08	0.033
	TPpred-ATMV^a^	0.772	0.054	0.027
	TPpred-LE	**0.879**	**0.420**	**0.391**

^a^The results are obtained by running their standalone programs

^b^PPTPP contains three variant approaches, including PPTPP-cls, PPTPP-prb, and PPTPP-fus, among which only the best results for each metric are reported

The compared methods are based on conventional machine learning, and they have extracted the hand-crafted manual features by integrating different properties. To erase the impact of the input features, we further compare TPpred-LE with one-versus-all RFs trained on one-hot and PSSM-encoded sequences of the training set as TPpred-LE. We trained a RF model for each function with a one-versus-all strategy. Besides, we constructed the input for RFs in two strategies: concatenating or averaging all the input residual vectors. The results are shown in Table 3, from which we can be shown that the one-versus-all RFs fail to effectively predict the therapeutic peptide functions, demonstrating the necessity of complex deep networks.

**Table 3:** The performance of TPpred-LE and one-versus-all RF classifier

Method	ACC_example_	F1_label_
TPpred-LE	0.536	0.422
RF-Concat^a^	0.060	0.060
RF-Avg^b^	0.068	0.047

^a^Concatenate all the input residual vectors as the sequence level input vectors

^b^Average all the input residual vectors as the sequence level input vectors

### Performance comparison between TPpred-LE and other multi-label classification methods

**Fig. 3:**
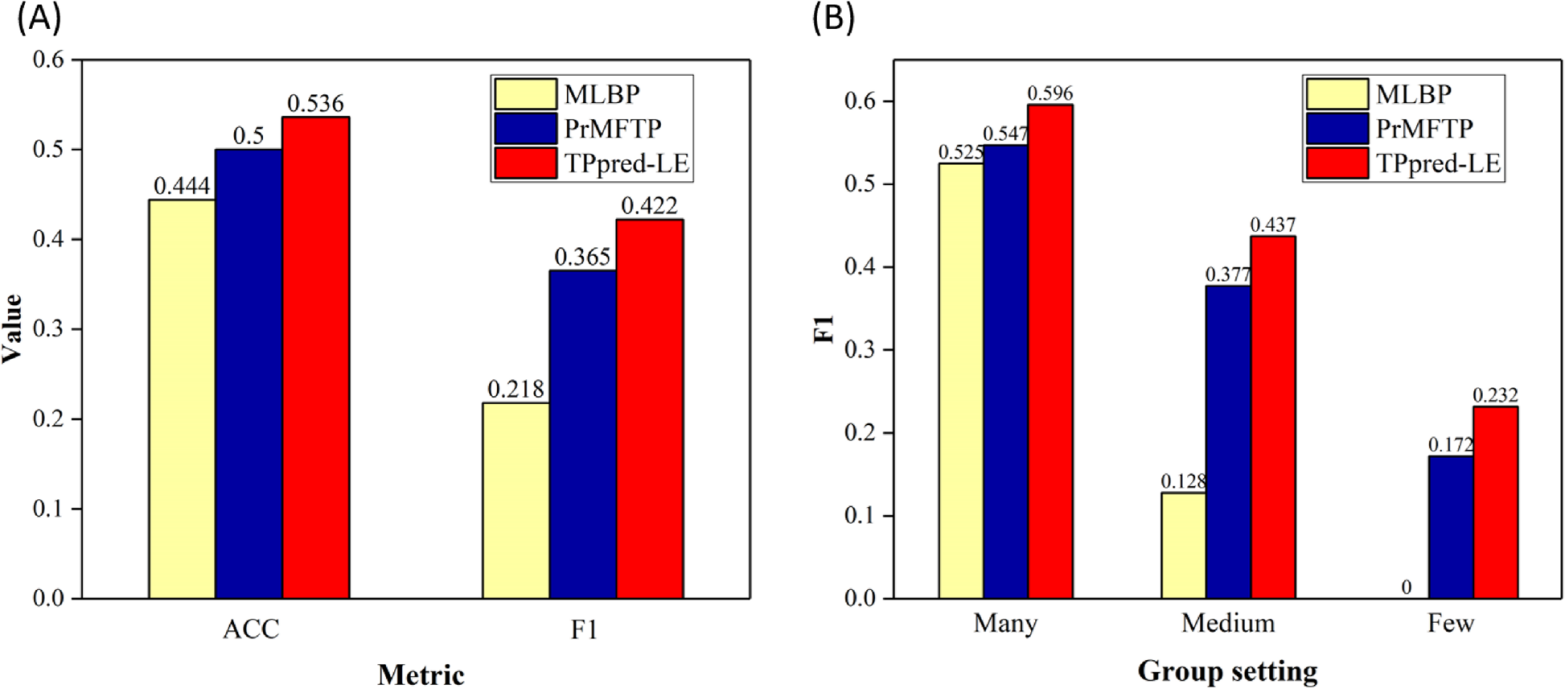
The performance of TPpred-LE, MLBP, and PrMFTP on the independent dataset. **A** The overall performance of the three methods on the independent datasets. **B** The $F{1}_{label}$ scores of the three methods for each function group

To further evaluate the performance of TPpred-LE, we compare TPpred-LE with MLBP [12] and PrMFTP [13], which are multi-label classification models for multi-functional peptide identification. To fairly and comprehensively evaluate the performance of TPpred-LE and the other methods, we retrain the other methods and evaluate them on ${\mathbb{S}}_{benchmark}$ (cf. Equation 1). The results are shown in Fig. 3A, from which we can see that TPpred-LE outperforms the other methods in all metrics. Figure 3B shows that MLBP and PrMFTP achieve lower performance on medium-shot functions, and MLBP even fails to predict the few-shot functions. In contrast, TPpred-LE achieves stable performance in all groups. Therefore, TPpred-LE is a useful tool for multi-label functional therapeutic peptide prediction.

### TPpred-LE can predict new therapeutic peptide functions with limited labeled training data

In previous therapeutic peptide prediction studies, there is an assumption that all peptide sequences are comprehensively labeled. However, the assumption hardly holds in reality [22, 23]. With the development of therapeutic peptide function analysis methods, more and more potential functions of therapeutic peptides are discovered in the future, which means that the currently known training data may only contain limited functions being annotated. Therefore, it is essential and desired to predict the newly detected therapeutic peptide functions with the limited labeled data for training. The limited labeled data means part of the positive samples are mislabeled as the negative samples for a function. For example, a sequence with AMP and ACP functions is only marked as AMP, which is called mislabeled. In this regard, to simulate this real world application, we construct a series of training and validation datasets by randomly removing the labels with the *weak label ratio* (WL ratio) [22] varying from 50 to 90% with 10% as the interval. The detailed construction steps are described in Additional file 2: Supplementary Material S2 [22].

**Fig. 4:**
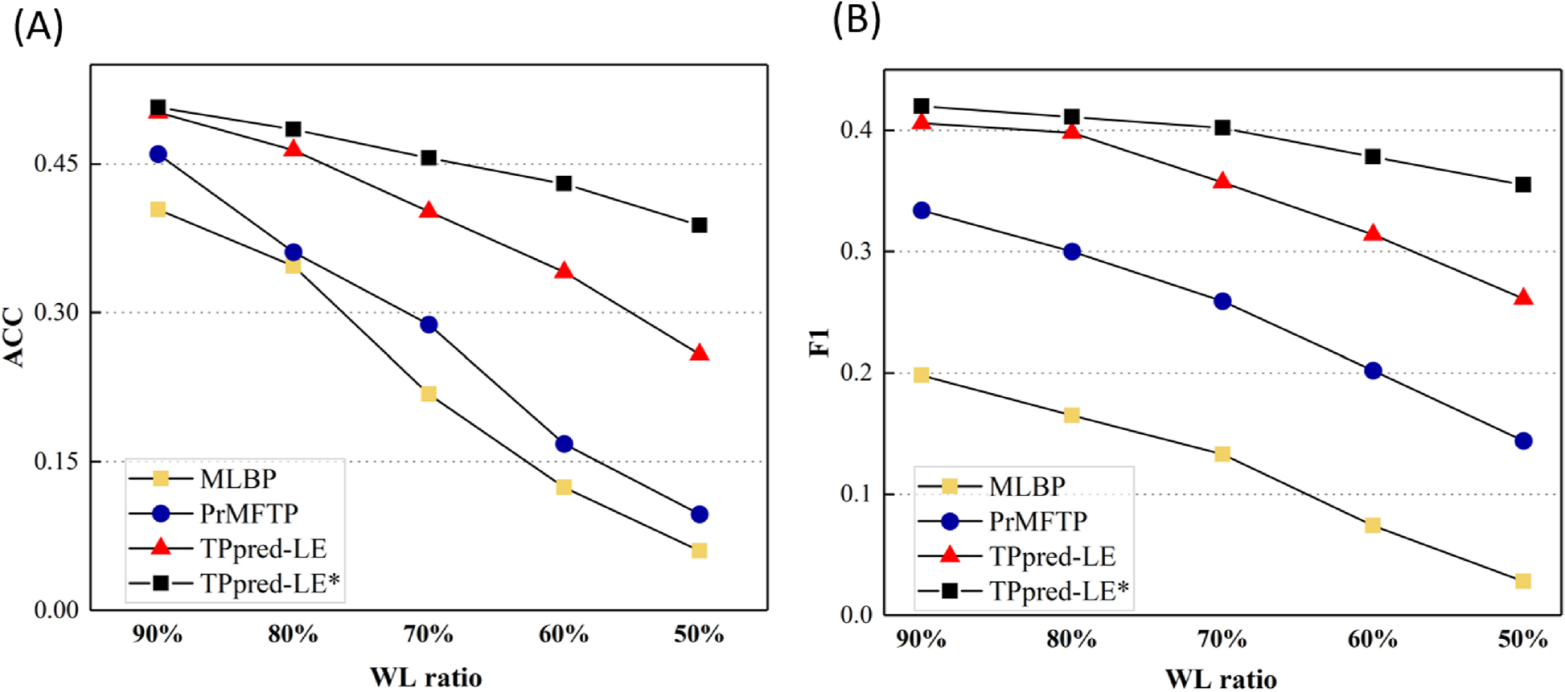
The performance of TPpred-LE*, TPpred-LE, MLBP, and PrMFTP on the independent test dataset. The *x*-axis indicates the training and validation data with different WL ratios

The performance of TPpred-LE* (TPpred-LE with MCRT), TPpred-LE, MLBP, and PrMFTP on these datasets with various WL ratios are shown in Fig. 4, from which we can see the following: (i) both the TPpred-LE* and TPpred-LE consistently outperform MLBP and PrMFTP on all the datasets; (ii) TPpred-LE* achieves the best performance by using the MCRT, indicating that the square root resampling plays a key factor in reducing the likelihood of selecting mislabeled samples. Therefore, the square root resampling strategy can improve the robustness of the model when there exist mislabeled samples. These results demonstrate that TPpred-LE is a useful for method for analyzing the newly detected therapeutic peptide functions with limited labeled data.

### Visualization of the attentions

**Fig. 5:**
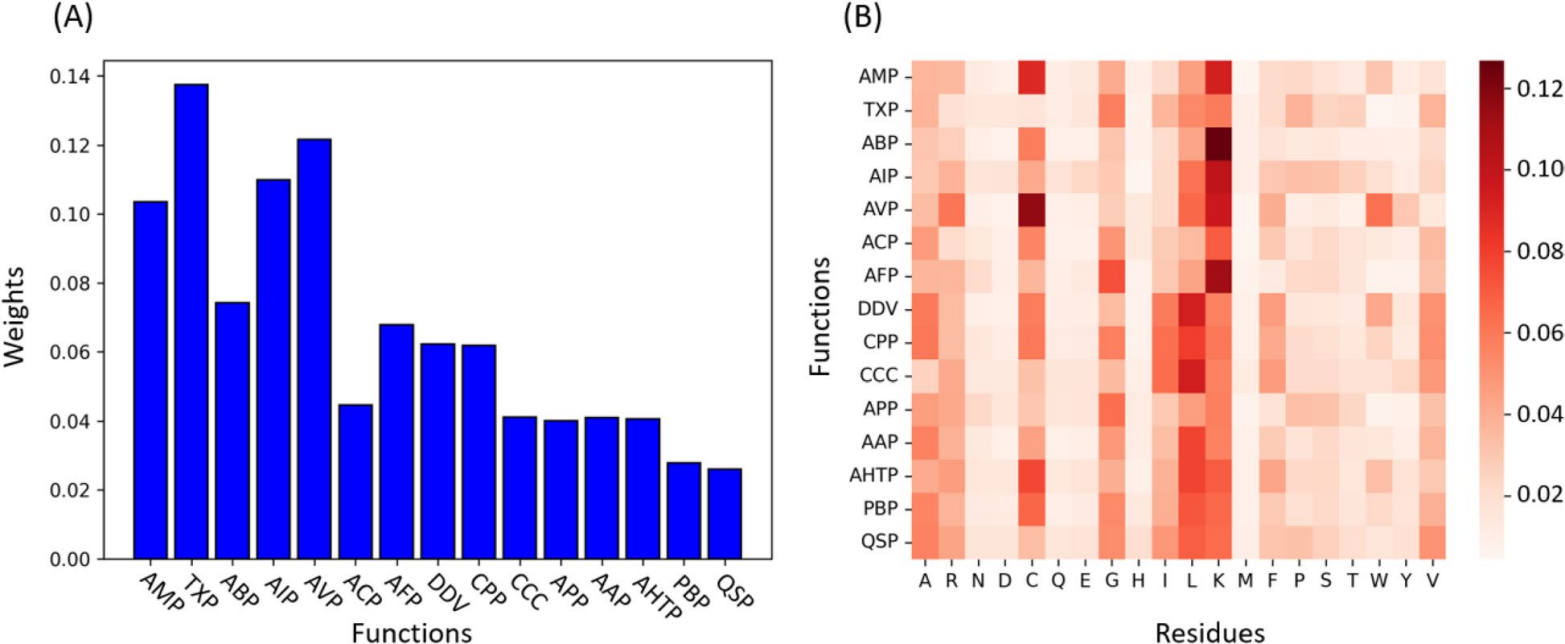
The averaged received attention weights for all functions and residues on the independent test dataset. The larger the weight, the more attention the model pays to that class during the prediction process. **A** is computed by averaging all function-function attention weights. **B** is computed by averaging all function-residue attention weights

To investigate the role of three types of attention in the model, we visualize the learned attention weights in the last layer of the Transformer encoder and decoder. We visualize the overall received attention weights for all functions and residues. The results are illustrated in Fig. 5. The weights distribution in Fig. 5A closely resembles the distribution in Fig. 3B. It shows that functions with larger quantities tend to have better prediction performance, so that they are likely to receive more attention. Figure 5B shows the overall function-residue functions. We can see that different functions are likely to have distinct preferences for the residues in the prediction process.

**Fig. 6:**
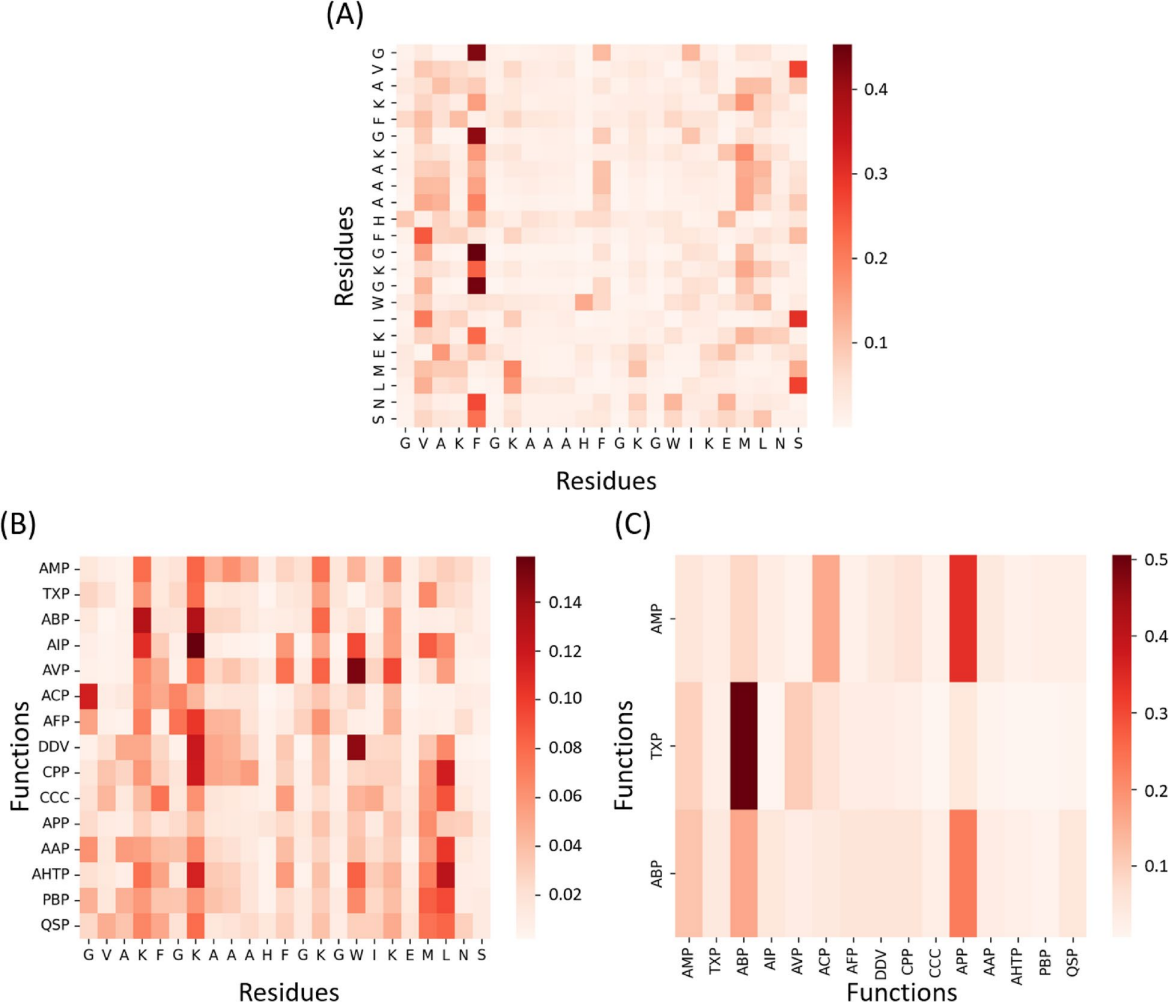
Visualization of three types of attentions for peptide “GVAKFGKAAAHFGKGWIKEMLNS.” Each row represents the attention weights of the current element (*y*-axis) towards the target elements (*x*-axis). **A** Residue-residue attention. **B** Function-residue attention. **C** Function-function attention

Furthermore, we focus on a single peptide sequence to visualize the three types of attentions. We take the peptide “GVAKFGKAAAHFGKGWIKEMLNS” as an example, which has the functions of AMP, TXP, and ABP. The weights of three types of attention are shown in Fig. 6. We can see that different residues and functions are likely to pay attention to different regions (residues) by using the sequential information in Fig. 6A and B. The function-function attention shown in Fig. 6C suggests the prediction process for its functions of AMP, TXP, and ABP. When predicting AMP, TPpred-LE pays more attention to ABP, ACP, APP, and so on. In other words, TPpred-LE utilizes the information from other functions to predict the AMP function for this sequence. The prediction processes of the other functions are in the same way. Therefore, TPpred-LE can leverage the relationship information among functions and residues to enhance the ability of multi-functional therapeutic peptides.

## Discussion

The aforementioned results reveal limitations in the predictive capabilities of current methods for therapeutic peptide function prediction. On one hand, the binary classification techniques focus on specific peptide functions, while overlooking the relationship information among different peptide functions. These methods frequently yield a high false-positive rate, resulting in lower precision. On the other hand, the existing multi-label classification-based methods still fail to explicitly employ the relationship information, which leads to unsatisfactory accuracy, particularly when dealing with a limited number of training samples.

TPpred-LE is an innovative approach designed for the prediction of multifunctional therapeutic peptides, which incorporates the relationship information between different peptide functions effectively. This method utilizes the encoder and decoder to learn the correlation information among residues and functions to improve the prediction ability as shown in the ablation experiment. Furthermore, TPpred-LE benefits from the integration of the attention mechanism, which allows for the straightforward visualization of attention weights for three different types. The three difference weight types improve the performance of TPpred-LE. Finally, we introduced the label missing problem in the therapeutic peptide function prediction field and proposed the MCRT algorithm to solve it. The study on the limited training labeled data is promising to predict the function more comprehensively. There still exist some limitations in the TPpred-LE. For example, TPpred-LE’s reliance on deep neural networks demands a substantial volume of training samples to effectively learn patterns. In the future, we are planning to incorporate the pre-trained models to improve the performance on therapeutic peptide prediction.

## Conclusions

In this paper, we propose a novel method called TPpred-LE for therapeutic peptide function prediction. Compared with the other existing computational methods, TPpred-LE has the following advantages: (i) it accurately and comprehensively predicts the 15 different therapeutic peptide functions; (ii) it incorporates label embedding and function-specific classifiers to measure the correlation relationship and the specificity relationship among peptide functions, respectively; (iii) it is able to stably detect the newly detected therapeutic peptide functions with limited labeled data by introducing the MCRT algorithm; and (iv) its web server is constructed, only requiring the peptide sequences in FASTA format as inputs.

## Methods

### Benchmark dataset

**Fig. 7:**
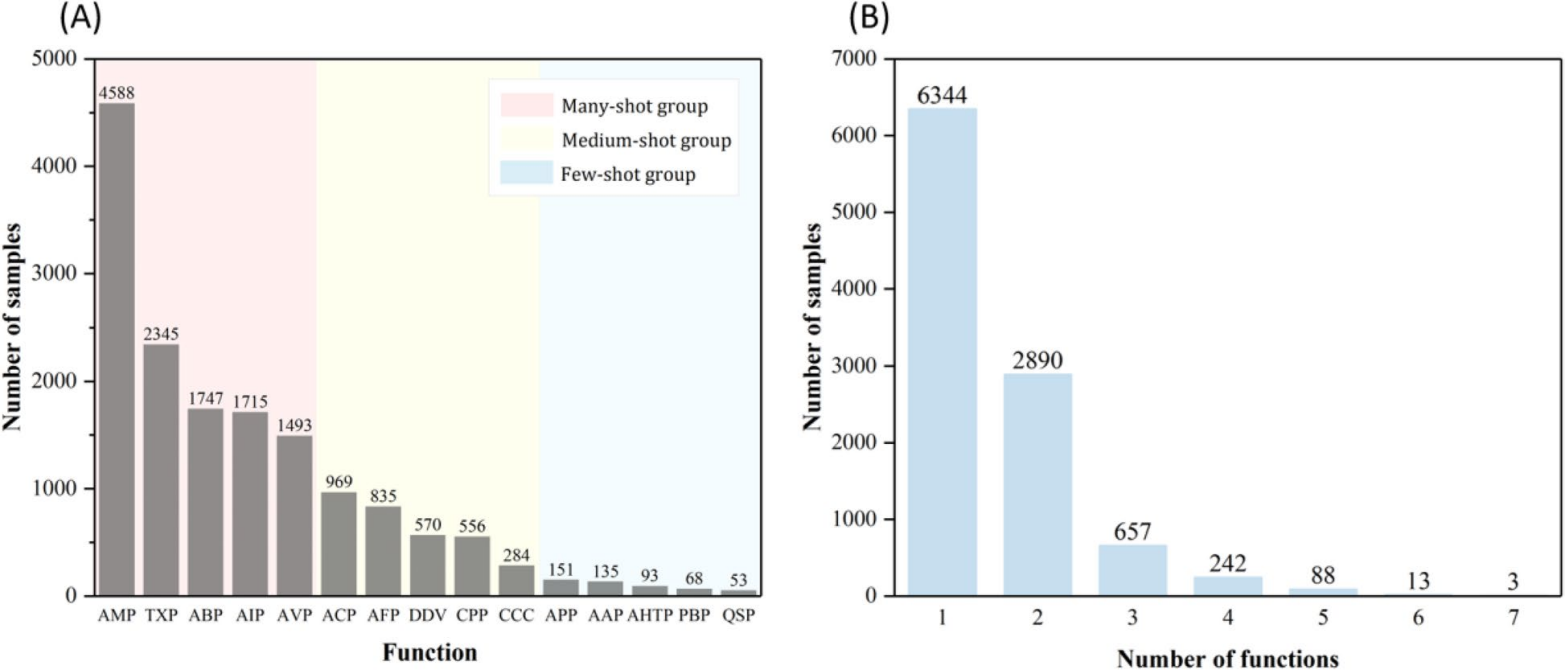
Function distribution of the samples in ${\mathbb{S}}_{benchmark}$. **A** The distribution of the number of samples in each function in ${\mathbb{S}}_{benchmark}$. **B** The distribution of the number of functions assigned to each sample in ${\mathbb{S}}_{benchmark}$

In this study, we constructed a comprehensive benchmark dataset with 15 different therapeutic peptide functions, including AMP, TXP, ABP, AIP, AVP, ACP, AFP, DDV, CPP, CCC, APP, AAP, AHTP, PBP, and QSP. They were derived from SATPdb [4], PEPred-Suite [9], DRAMP 2.0 [24], Basith S’s review [25], and AntiCP 2.0 [26]. The details were listed in Additional file 3: Table S3 [4, 9, 24–26]. The benchmark dataset can be represented as:

1 $${\mathbb{S}}_{benchmark}={\mathbb{S}}_{\mathrm{AMP}}\cup {\mathbb{S}}_{\mathrm{TXP}}\cup \dots \cup {\mathbb{S}}_{\mathrm{QSP}}$$

where ${\mathbb{S}}_{\mathrm{AMP}},{\mathbb{S}}_{\mathrm{TXP}},\dots ,{\mathbb{S}}_{\mathrm{QSP}}$ are the subsets containing the specific therapeutic peptide functions. Sequences sharing similarity higher than 90% in each subset were removed [27–30] by using CD-HIT [31]. Finally, the benchmark dataset contains 10,237 unique sequences with one or more functions. The statistical information of the benchmark dataset is shown in Fig. 7. The detailed distribution of different multi-functions and their relationship is shown in Additional file 4: Fig S1.

As illustrated in Fig. 7, the number of samples with different functions is obviously imbalanced, following a long-tail distribution [23]. In order to better examine the performance variations across functions with different numbers of samples, we divide all the 15 functions into 3 groups according to their number of samples [15, 32, 33]: many-shot group (more than 1000 samples), medium-shot group (200 ~ 1000 samples), and few-shot group (less than 200 samples). To train and evaluate models, we randomly split the ${\mathbb{S}}_{benchmark}$ into training dataset, validation dataset, and independent test dataset roughly with the ratio of 8:1:1. The homology similarity between training dataset and independent test dataset as well as the validation dataset is less than 90% for each function.

### Sequence embedding and label embedding

The embedding modules in TPpred-LE learn the discriminative representations of sequences and the therapeutic peptide functions.

Firstly, the input sequences and all the functions are embedded as numerical vectors. One-hot encoding [34] and position-specific scoring matrix (PSSM) [35] are adopted to encode the peptide sequences. One-hot is a binary vector encoding the amino acid in each position into a vector with the dimension of 20 to represent the composition information of the sequence. PSSM captures the evolutionary information of the sequence and encodes each amino acid into a vector with the dimension of 20. We generate the PSSMs through the multiple sequence alignments (MSAs) by using PSI-BLAST [35] (‘-num_iterations 3 -evalue 0.01’) to search against the NR database [36]. Finally, the feature vector of each sequence is obtained by concatenating the two features. The functions are represented as one-hot encoding, and each peptide function class is represented as a vector with the dimension of 15.

For a given sequence, the length of the input sequence is *L*, which is fixed as 50 in this study. If the length of the sequence is less than $50$, we pad it with zeros at the end of the sequence, while if the length of the sequence exceeds $50,$ two sub-sequences with length of 25 from its N-terminal and C-terminal are extracted and concatenated [37]. We have also tested another sequence truncating strategy, which only extracts the sub-sequence from the sequence beginning side (N-terminal) as [5] or most of the natural language processing (NLP) tasks [38] generally do. The performance results listed in Additional file 3: Table S4 show that the above two truncating strategies are comparable to each other. Since the majority of sequences in the benchmark dataset have a length of less than 50 (see Additional file 4: Fig S2), the sequence truncating strategy only needs to be applied to a small number of sequences. Therefore, the choice of truncation strategy has minimal impact on this study, and we just chose the N-terminal and C-terminal. Moreover, as most of the sequences in our benchmark dataset have at least 10 amino acids after performing homology reduction, the sequences with lengths less than 10 are likely to have a bias prediction. We limited the minimum length of the input to 10 in our webserver.

An encoded sequence is represented as ${\mathbf{X}}^{s}={\left\{{x}_{i}^{s}\right\}}_{i=1}^{L}\in {\mathbb{R}}^{L\times 40}$, and the encoded function set is defined as ${\mathbf{X}}^{t}={\left\{{x}_{i}^{t}\right\}}_{i=1}^{C}\in {\mathbb{R}}^{C\times C}$, where $C$ is the number of all therapeutic peptide functions. In this study, *C* is set as 15.

**Fig. 8:**
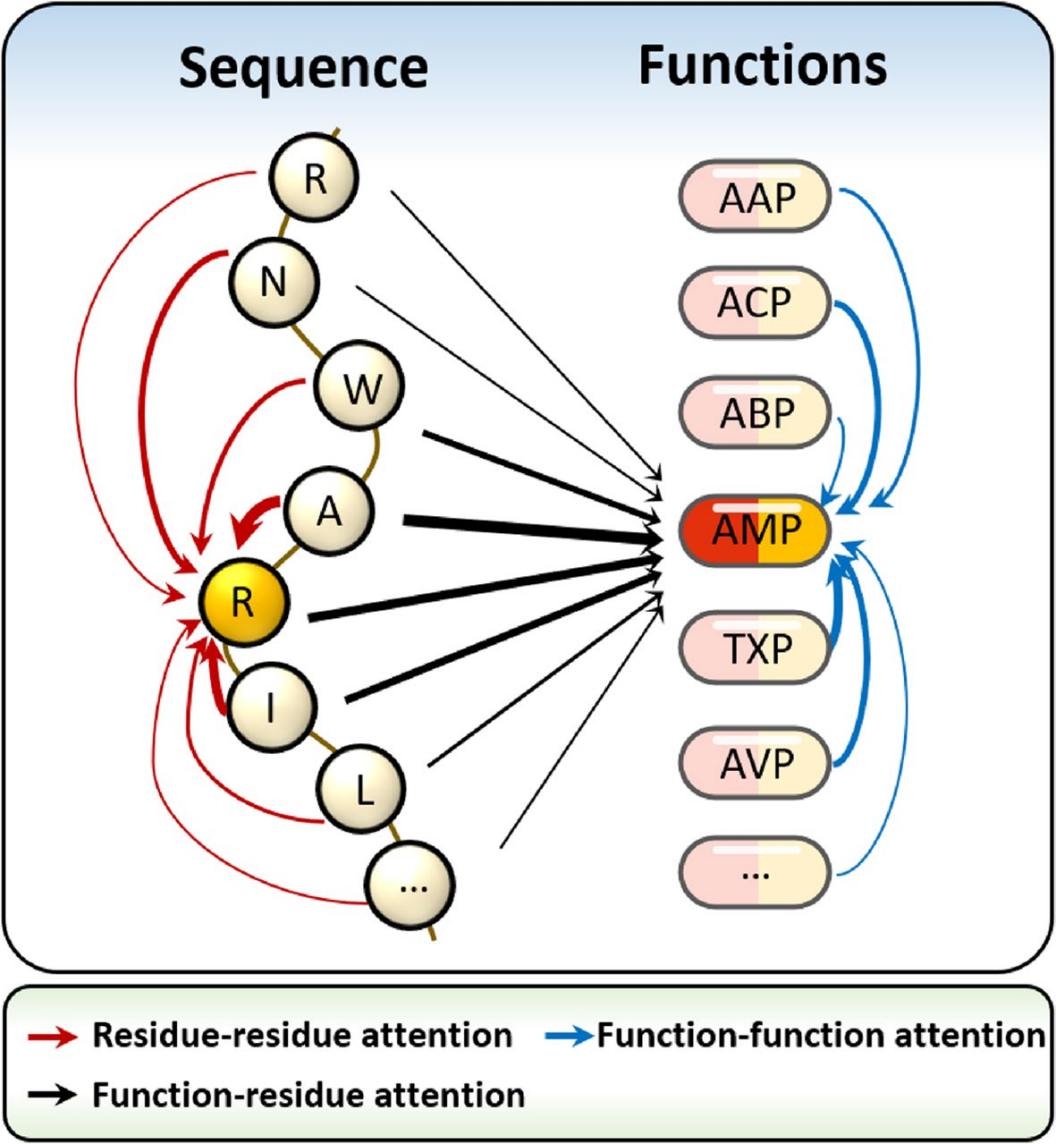
Three types of attention employed by TPpred-LE

We adopt Transformer [39] to learn the representation of sequences and functions. The self-attention mechanism [39] in Transformer allows the model to focus on the prediction related regions. The attentions in Transformer can be divided into three types according to their different roles: (i) residue-residue attention, (ii) function-function attention, and (iii) function-residue attention as shown in Fig. 8. The residue-residue attention has been used in the other studies to learn the representation of protein sequences [40, 41]. The correlation relationship among different therapeutic peptide functions is ignored by the exiting methods. Therefore, we explore the correlation relationship among therapeutic peptide functions based on label embedding methodology [42–45] through the Transformer decoder. There are two attentions in the label embedding module, including function-function attention and function-residue attention. The function-function attention allows each function updates its representation according to the information from the other functions, while the function-residue attention integrates the information between residues and functions. The mathematical description of the all attentions in Transformer can be represented as [39]:

2 $$Attention\left(\mathbf{Q},\mathbf{K},\mathbf{V}\right)=softmax\left(\frac{\mathbf{Q}{\mathbf{K}}^{T}}{\sqrt{{d}_{model}}}\right)\mathbf{V}$$

where ${d}_{model}$ represents the hidden dimension of the model. $\mathbf{Q}$, **K**, and **V** are the query, key, and value matrices, respectively.

Multi-head attention mechanism allows the model to attend to information from different perspectives [37, 39, 41] adopted in [39]:

3 $$MultiHeadAttention=\mathrm{ Concat}\left({\mathrm{head}}_{1}, {\mathrm{head}}_{2},\dots ,{\mathrm{head}}_{h}\right){\mathbf{W}}^{O}$$

4 $$hea{d}_{i}=Attention\left(\mathbf{X}{\mathbf{W}}_{i}^{Q},\mathbf{X}{\mathbf{W}}_{i}^{K},\mathbf{X}{\mathbf{W}}_{i}^{V}\right)$$

where the $\mathbf{X}$ represents the input of the encoder or decoder. ${\mathbf{W}}_{i}^{Q}, {\mathbf{W}}_{i}^{K},{\mathbf{W}}_{i}^{V}\in {\mathbb{R}}^{{d}_{model}\times {d}_{model}}$ are the projection matrix of query, key, and value, respectively. The $h$ represents the number of attention heads. ${\mathbf{W}}^{O}\in {\mathbb{R}}^{h{d}_{model}\times {d}_{model}}$ transforms the dimension of the concatenated vectors into the feature space with the dimension of ${d}_{model}$.

The encoder takes ${\mathbf{X}}^{s}$ as input, and the decoder takes ${\mathbf{X}}^{t}$ as input. The function representation $\mathbf{Z}={\left\{{z}_{i}\right\}}_{i=1}^{C}\in {\mathbb{R}}^{C\times {d}_{model}}$ is learned by Transformer [39]:

5 $$\mathbf{Z}=Transformer\left({{f}_{enc}(\mathbf{X}}^{s}\right)+\mathbf{P}\mathbf{E}, {{f}_{dec}(\mathbf{X}}^{t}))$$

where ${f}_{enc}(\cdot )$ and ${f}_{dec}(\cdot )$ are linear projection layers converting the low-dimensional input vectors into the feature space with the high dimension of ${d}_{model}$. $Transformer(\cdot )$ represents the complete Transformer neural network as shown in Fig. 2. Please refer to [39] for more details of the Transformer.

The positional encodings ($\mathbf{P}\mathbf{E})$ are added into the input sequence embedding to preserve the residue order information [39]:

6 $$\mathbf{PE}\left(pos,2i\right)=sin\left(\frac{pos}{1000^{2i/d^{\prime}_{model}}}\right)$$

7 $$\mathbf{PE}\left(pos,2i+1\right)=cos\left(\frac{pos}{1000^{2i/d^{\prime}_{model}}}\right)$$

where $pos$ indicates the position of the amino acid in the sequence ($0\le pos\le L-1$) and $0\le i<{d}_{model}^{\mathrm{^{\prime}}}/2$. In this study, ${d}_{model}^{\mathrm{^{\prime}}}$ is equal to ${d}_{model}$.

### Function-specific classifiers

For each sequence, the output of the embedding modules is a function representation matrix $\mathbf{Z}$. To transform the high dimensional representation $\mathbf{Z}$ into the output space, a common approach is to simply add a single linear layer:

8 $$\widehat{y}=sigmoid(\mathbf{Z}{w}_{single}+{b}_{single})$$

where ${w}_{single}\in {\mathbb{R}}^{{d}_{model}}$ and ${b}_{single}\in {\mathbb{R}}$, which are shared with all functions. The $\widehat{y}\in {\mathbb{R}}^{C}$ is the predicted probabilities for all therapeutic peptide functions.

**Fig. 9:**
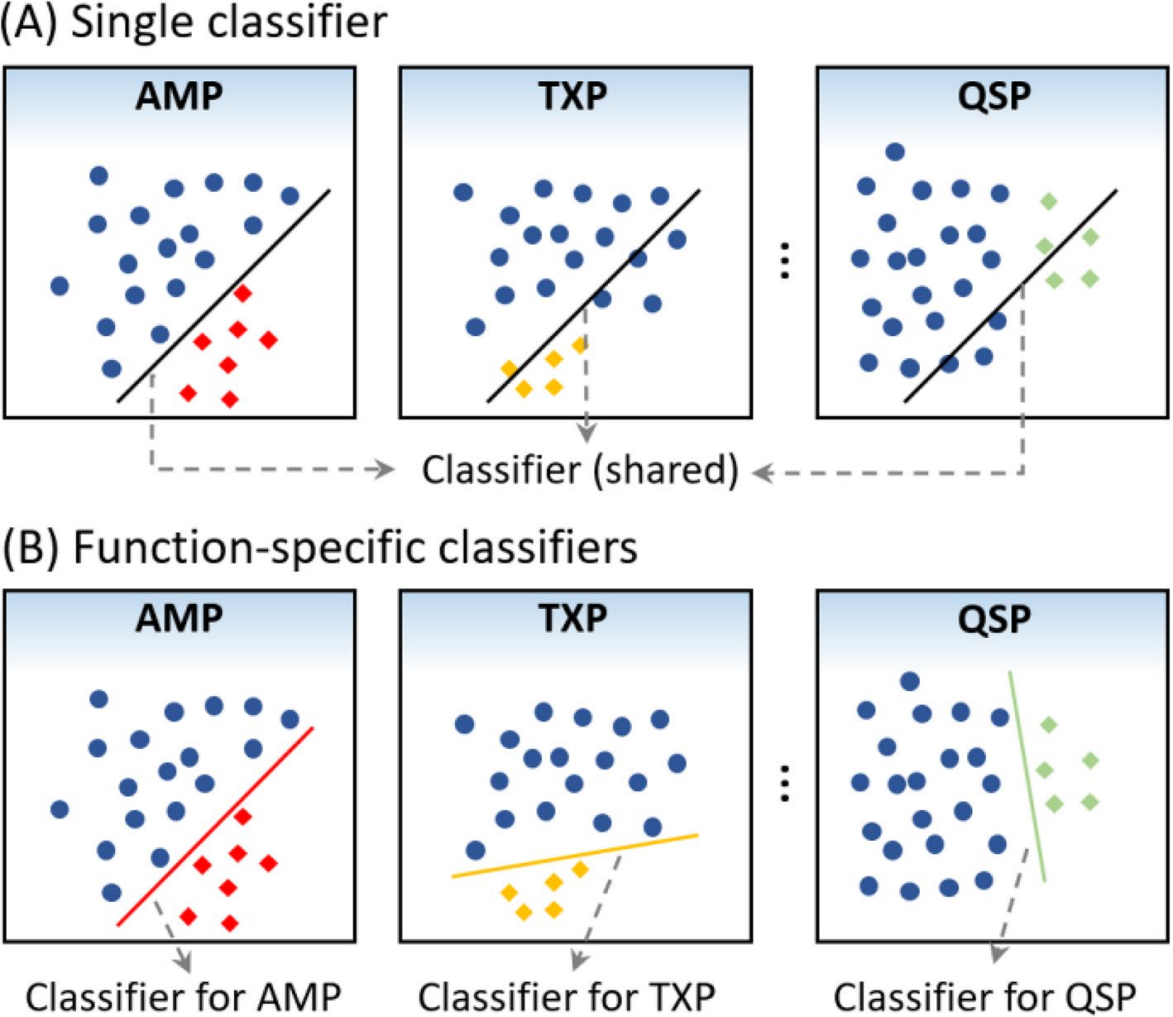
Comparison between single classifier and function-specific classifiers. **A** When using the single classifier, all the functions share the same classifier. **B** When using the function-specific classifiers, each function will learn an independent classifier according to its distribution of the representation vectors

However, this approach fails to capture the specificity of different therapeutic peptide functions (see Fig. 9A). Therefore, for each therapeutic peptide function, we design an independent classifier to learn an independent decision boundary for each function according to the distinct feature distribution (see Fig. 9B). In addition, each classifier can be regulated independently without interfering the classifiers for the other functions, which allows us to train all classifiers in a multi-label classification approach; meanwhile, we can adjust each classifier in a binary classification manner, demonstrating its scalability. The prediction process of TPpred-LE based on function-specific classifiers can be represented as:

9 $${\widehat{y}}_{i}={sigmoid(w}_{i}{\cdot z}_{i}+{b}_{i}), i\in [1, C]$$

where $${w}_{i}\in {\mathbb{R}}^{{d}_{model}}$$ and $${b}_{i}\in {\mathbb{R}}$$.

Finally, we obtain the predicted functions for each peptide with the threshold of 0.5.

### Multi-label classifier retraining (MCRT)

In order to predict the new therapeutic peptide functions with limited labeled data, we propose the multi-label classifier retraining (MCRT) strategy for detecting new functions with limited labeled data.

Classifier retraining (cRT) has been confirmed to be an effective approach for long-tailed multi-class classification [15], which learns the representation using the original imbalanced data and employs the resampled balanced training data to retrain the classifier with the representation module keeping fixed. In this study, we extend the cRT approach to the multi-label classification task so as to enhance the prediction ability of TPpred-LE for detecting new functions with the limited labeled data.

Benefiting from the scalability of the function-specific classifiers, we treat the model as $C$ binary classifiers and retrain each classifier separately. For each classifier, we resample the training dataset to get the corresponding balanced training dataset with $N$ samples based on the bootstrap strategy [46]. The square root sampling strategy [47, 48] is used in this study. The sampling probability ${p}_{cj}$ is defined as [15]:

10 $${p}_{cj}=\frac{\sqrt{{n}_{cj}}}{\sqrt{{n}_{cj}}+\sqrt{N-{n}_{cj}}}$$

where $c\in \{AMP, TXP,\dots ,QSP\}$ represents a specific function, $j\in \left\{positive, negative\right\}, {n}_{cj}$ is the number of positive or negative training samples of a specific class, and $N$ is the number of all training samples.

MCRT retrains each classifier with the resampling training dataset for each function. When retraining the classifier $c$, we feed the corresponding sampled training dataset and freeze the embedding modules and the classifiers for the other functions. As a result, the prediction of other functions will not be affected.

### The model implementation

In TPpred-LE, each function will be projected into a distinct output space due to the independency of each function-specific classifier, which will adversely affect the label embedding process. Therefore, we utilize two training steps to train TPpred-LE. In the first training step, the single classifier is used to learn the embedding in the same output space so as to extract the correlation information among labels. In the second training step, we replace the single classifier with the function-specific classifiers to train the model with the label embedding module keeping fixed, and each classifier will obtain a distinct decision boundary according to its specificity information. The detailed training process is shown in Algorithm 1. Besides, the training process of TPpred-LE based on MCRT is shown in Algorithm 2. The ${E}_{seq}, {E}_{func}, {F}_{single},{F}_{specific}$ represent the learnable parameters in the sequence embedding module, the label embedding module, the single classifier, and the specific classifiers, respectively. Binary cross entropy loss [49] is used to measure the gap between the ground truth labels and the prediction [49]:

11 $$Loss\left({\widehat{y}}_{i}, {y}_{i}\right)={\sum }_{j=1}^{C}{y}_{ij}\cdot log{\widehat{y}}_{ij}+(1-{y}_{ij})\mathrm{log}(1-{\widehat{y}}_{ij})$$

where ${y}_{ij}\in {\mathbb{R}}$ is the ground truth label, and ${\widehat{y}}_{ij}\in {\mathbb{R}}$ is the prediction probability corresponding to function $j$ for the sample $i$. AdamW [50] algorithm is used to optimize the trained parameters. Each training step runs 30 epochs. The hyperparameters are determined by the grid search strategy according to the minimum of the validation loss in each training setting. The detailed hyperparameters and their optimal values of TPpred-LE are listed in Additional file 3: Table S5. In this work, each experiment is run for 5 times with different random seeds, and the average results are reported so as to ensure the reliability.

### Evaluation metrics

For multi-label classification, the evaluation metrics are generally categorized into two groups [16]: example-based metrics and label-based metrics. Example-based metrics are the averaged measure for all samples. Label-based metrics consider each function has equal importance and perform averaging among all functions. The previous works [12, 13, 51] only reports the example-based metrics ignoring the label-based metrics. As a result, the prediction ability for the functions with fewer samples cannot be clearly illustrated, such as the functions in few-shot groups as shown in Fig. 6. Therefore, we comprehensively evaluate our method by using two types of metrics:

12 $$AC{C}_{example}=\frac{1}{N}{\sum }_{i=1}^{N}\frac{\Vert {L}_{i}\cap {\widehat{L}}_{i}\Vert }{\Vert {L}_{i}\cup {\widehat{L}}_{i}\Vert }$$

13 $$F{1}_{label}=\frac{1}{C}{\sum }_{i=1}^{C}F{1\mathrm{measure}}_{i}$$

where $AC{C}_{example}$ is used as the example-based metric following [12, 13, 52], ${L}_{i}$ is the ground truth label set, and ${\widehat{L}}_{i}$ is the predicted label set. When calculating label-based metrics, we split the muti-label classification task into multiple binary classification tasks and average them to obtain the final metrics. $F{1}_{label}$ (macro-F1) is used as the measure of the label-based metric. We also utilize the binary classification metrics to evaluate the binary prediction performance, including AUC [17], MCC [18], F1 [19], and RkCC [20].

**Figure:**
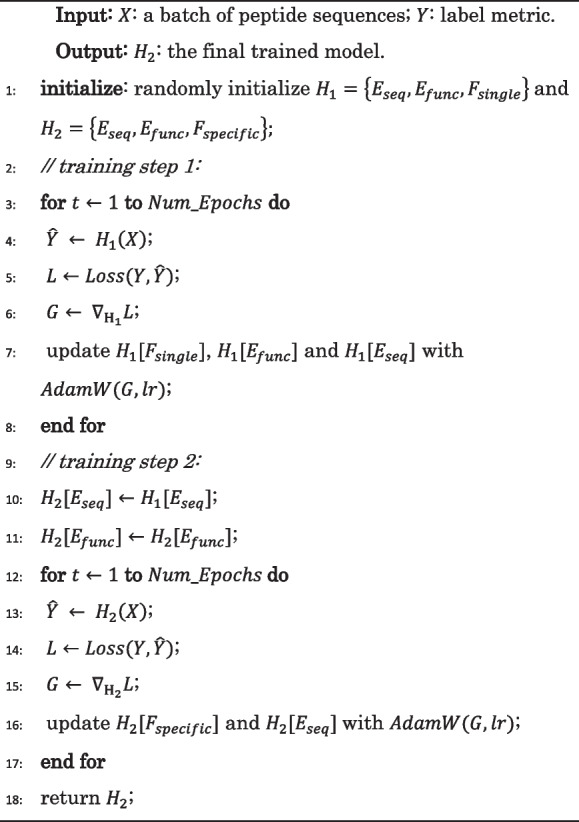
**Algorithm 1.** The training steps of TPpred-LE without MCRT

**Figure:**
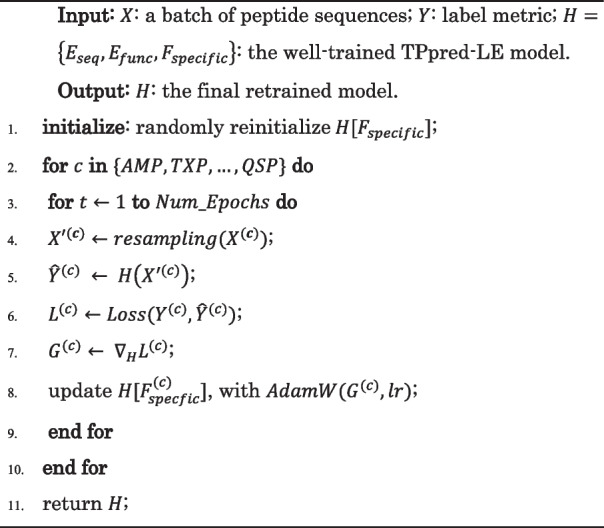
**Algorithm 2.** The training steps of TPpred-LE with MCRT

### Supplementary Information

**Additional file 1:** **Supplementary Material S1.** The calculation of the Pearson’s correlation coefficient.

**Additional file 2:** **Supplementary Material S2.** The construction of the limited labelled datasets.

**Additional file 3:** **Table S1.** The precision scores of various methods for predicting eight therapeutic peptide functions on the independent dataset. **Table S2.** The performance of TPpred-LE for predicting 15 therapeutic peptide functions on the independent dataset. **Table S3.** The statistical information of the 15 therapeutic peptide functions. **Table S4.** The performance comparison of two strategies for truncating the sequences with length exceeding 50. **Table S5.** The search space for hyperparameters and their optimal values used in TPpred-LE.

**Additional file 4:** **Fig S1.** The distribution of different multi-functions and their relationship. **Fig S2.** The length distribution of the benchmark dataset.

## Data Availability

The TPpred-LE webserver is accessible at http://bliulab.net/TPpred-LE/ [53]. The data and codes utilized in this study is available at http://bliulab.net/TPpred-LE/data/ [53] and https://github.com/HongWuL/TPpred-LE [54] respectively. The source codes reach a bronze standard of reproducibility.

## Abbreviations

RF: Random forest
AMP: Anti-microbial peptide
TXP: Toxic peptide
ABP: Anti-bacterial peptide
AIP: Anti-inflammatory peptide
AVP: Anti-viral peptide
ACP: Anti-cancer peptide
AFP: Anti-fungal peptide
DDV: Drug delivery vehicle peptide
CPP: Cell-penetrating peptide
CCC: Cell-cell communication peptide
APP: Anti-parasitic peptide
AAP: Anti-angiogenic peptide
AHTP: Anti-hypertensive peptide
PBP: Polystyrene surface-binding peptide
QSP: Quorum sensing peptide
ACC _example_: Example-based accuracy
F1_label_: Label based F1-score
AUC: The area under the ROC curve
MCC: Matthews’s correlation coefficient
Rkcc: K-category correlation coefficient
cRT: Classifier retraining approach
MCRT: Multi-label classifier retraining approach
