# Supplementary material for: TPpred-LE: therapeutic peptide function prediction based on label embedding
Source: BMC Biol. 2023 Oct 31;21:238. doi: 10.1186/s12915-023-01740-w (PMC10617231; doi:10.1186/s12915-023-01740-w)
Supplement: Supplementary file 1 — Additional file 1: Supplementary Material S1. The calculation of the Pearson’s correlation coefficient. [file 12915_2023_1740_MOESM1_ESM.docx]

**The calculation of the Pearson’s correlation coefficient**

The Pearson’s correlation coefficient is formulated as [21]:

| $\rho\left( x_{i},x_{j} \right)=\frac{cov(x_{i}, x_{j})}{\sigma_{x_{i}}\sigma_{x_{j}}}$ | (1) |
| --- | --- |

where $x_{i}$ and $x_{j}$ are the representation vectors. $cov(x_{i},x_{j})$ is the covariance for $x_{i}$ and $x_{j}$. $\sigma_{x_{i}}$ and $\sigma_{x_{j}}$ are the standard deviation for $x_{i}$ and $x_{j}$, respectively.

The correlation coefficient for the functions $i$ and $j$ in the training set is calculated by (1):

| $\rho_{ij}^{data}=\rho(\mathbf{Y}_{\cdot i}, \mathbf{Y}_{\cdot j})$ | (2) |
| --- | --- |

where $\mathbf{Y}\in\mathbb{R}^{N\times C}$ is the binary label matrix with $N$ training samples and $C$ functions, $\mathbf{Y}_{\cdot i}\in\mathbb{R}^{N}$ represents the label vectors of function $i$.

The correlation coefficient for the functions $i$ and $j$ learned by TPpred-LE is calculated by averaging the function correlation coefficients of all training samples (1):

| $\rho_{ij}^{model}=\frac{1}{N}\sum_{t=1}^{N} \rho\left( \mathbf{Z}_{i\cdot}^{(t)},\mathbf{Z}_{j\cdot}^{(t)} \right)$ | (3) |
| --- | --- |

where $\mathbf{Z}^{t}\in\mathbb{R}^{C\times d_{model}}$ is the function representation matrix for sample$t$ obtained by the label embedding module, and $\mathbf{Z}_{i\cdot}^{(t)}$ represents the representation vector for function $i$.
