# Supplementary material for: TPpred-LE: therapeutic peptide function prediction based on label embedding
Source: BMC Biol. 2023 Oct 31;21:238. doi: 10.1186/s12915-023-01740-w (PMC10617231; doi:10.1186/s12915-023-01740-w)
Supplement: Supplementary file 2 — Additional file 2: Supplementary Material S2. The construction of the limited labelled datasets. [file 12915_2023_1740_MOESM2_ESM.docx]

**The construction of the limited labelled datasets**

We construct five limited labelled datasets (training sets and validation sets) with the *weak label ratio* (WL ratio) [22] varying from 50% to 90% with 10% as the interval. The WL ratio is defined as [22]:

$$WL ratio= \frac{\|\bar{\mathbf{Y}}\left. \right\|_{1}}{\|\mathbf{Y}\left. \right\|_{1}}$$

where $\bar{\mathbf{Y}}\in\mathbb{R}^{N\times C}$ is the binary label matrix with mislabelled samples, and $\mathbf{Y}\in\mathbb{R}^{N\times C}$ is the complete binary label matrix.

In order to simulate the situation of several therapeutic peptides have unknown functions, we construct the $\bar{\mathbf{Y}}$ with five different WL ratios. The “one” elements in the $\bar{\mathbf{Y}}$ (defined as the positive label) are randomly set as “zero” (defined as the negative label). The independent dataset remains unchanged to represent the comprehensive new functions, which would be discovered in the future.
