# Supplementary material for: TPpred-LE: therapeutic peptide function prediction based on label embedding
Source: BMC Biol. 2023 Oct 31;21:238. doi: 10.1186/s12915-023-01740-w (PMC10617231; doi:10.1186/s12915-023-01740-w)
Supplement: Supplementary file 3 — Additional file 3: Table S1. The precision scores of various methods for predicting eight therapeutic peptide functions on the independent dataset. Table S2. The performance of TPpred-LE for predicting 15 therapeutic peptide functions on the independent dataset. Table S3. The statistical information of the 15 therapeutic peptide functions. Table S4. The performance comparison of two strategies for truncating the sequences with length exceeding 50. Table S5. The search space for hyperparameters and their optimal values used in TPpred-LE. [file 12915_2023_1740_MOESM3_ESM.docx]

**Table S1.** The precision scores of various methods for predicting eight therapeutic peptide functions on the independent dataset.

| **Function** | **Method** | **Precision** |
| --- | --- | --- |
| AAP | PEPred-Suite | 0.016 |
|  | PPTPP | 0.017 |
|  | TPpred-ATMV | 0.014 |
|  | TPpred-LE | **0.299** |
| ABP | PEPred-Suite | 0.235 |
|  | PPTPP | 0.233 |
|  | TPpred-ATMV | 0.228 |
|  | TPpred-LE | **0.457** |
| ACP | PEPred-Suite | 0.086 |
|  | PPTPP | 0.091 |
|  | TPpred-ATMV | 0.105 |
|  | TPpred-LE | **0.472** |
| AIP | PEPred-Suite | 0.107 |
|  | PPTPP | 0.099 |
|  | TPpred-ATMV | 0.114 |
|  | TPpred-LE | **0.605** |
| AVP | PEPred-Suite | 0.093 |
|  | PPTPP | 0.091 |
|  | TPpred-ATMV | 0.102 |
|  | TPpred-LE | **0.535** |
| CPP | PEPred-Suite | 0.077 |
|  | PPTPP | 0.076 |
|  | TPpred-ATMV | 0.075 |
|  | TPpred-LE | **0.501** |
| PBP | PEPred-Suite | 0.036 |
|  | PPTPP | 0.038 |
|  | TPpred-ATMV | 0.046 |
|  | TPpred-LE | **0.571** |
| QSP | PEPred-Suite | 0.022 |
|  | PPTPP | 0.017 |
|  | TPpred-ATMV | 0.014 |
|  | TPpred-LE | **0.599** |

**Table S2.** The performance of TPpred-LE for predicting 15 therapeutic peptide functions on the independent dataset.

| **Function** | **AUC** | **MCC** | **F1** | **Rkcc** |
| --- | --- | --- | --- | --- |
| AMP | 0.791 | 0.452 | 0.721 | 0.452 |
| TXP | 0.9 | 0.633 | 0.711 | 0.633 |
| ABP | 0.834 | 0.338 | 0.426 | 0.338 |
| AIP | 0.895 | 0.527 | 0.594 | 0.527 |
| AVP | 0.835 | 0.457 | 0.529 | 0.457 |
| ACP | 0.773 | 0.337 | 0.371 | 0.337 |
| AFP | 0.804 | 0.242 | 0.247 | 0.242 |
| DDV | 0.889 | 0.442 | 0.471 | 0.442 |
| CPP | 0.899 | 0.477 | 0.502 | 0.477 |
| CCC | 0.945 | 0.584 | 0.595 | 0.584 |
| APP | 0.698 | 0.05 | 0.053 | 0.05 |
| AAP | 0.745 | 0.278 | 0.285 | 0.278 |
| AHTP | 0.708 | -0.005 | 0 | -0.005 |
| PBP | 0.934 | 0.443 | 0.43 | 0.443 |
| QSP | 0.879 | 0.42 | 0.391 | 0.42 |

**Table S3.** The statistical information of the 15 therapeutic peptide functions.

| **Function** | **Number** | **Source** |
| --- | --- | --- |
| AMP | 4588 | [4, 24] |
| TXP | 2345 | [4] |
| ABP | 1747 | [4, 9] |
| AIP | 1715 | [7, 25] |
| AVP | 1493 | [4, 9] |
| ACP | 969 | [4, 9, 25, 26] |
| AFP | 835 | [4] |
| DDV | 570 | [4] |
| CPP | 556 | [9, 25] |
| CCC | 284 | [4] |
| APP | 151 | [4] |
| AAP | 135 | [9] |
| AHTP | 93 | [4, 25] |
| PBP | 68 | [9] |
| QSP | 53 | [9, 25] |

**Table S4.** The performance comparison of two strategies for truncating the sequences with length exceeding 50.

| **Padding method** | **ACC_example_** | **F1_label_** |
| --- | --- | --- |
| N-25 + C-25 truncating (this study) ^a^ | **0.536** | 0.422 |
| N-50 truncating ^b^ | 0.534 | **0.425** |

^a^ Extract and concatenate two sub-sequences with length of 25 from its N-terminal and C-terminal.

^b^ Extract the sub-sequence with length of 50 from the N-terminal.

**Table S5.** The search space for hyperparameters and their optimal values used in TPpred-LE.

| **Hyperparameters** | **Description** | **Search space** | **Optimal values** |
| --- | --- | --- | --- |
| b | Batch size | [64, 128, 256] | 64 |
| lr | Learning rate | [1e-2, 1e-3, 1e-4, 1e-5] | 1e-4 |
| drop | Dropout rate | [0.1, 0.3, 0.5] | 0.1 |
| $d_{model}$ | Hidden dimension of Transformer | [64, 128, 256] | 256 |
| $h$ | Number of attention heads | [1, 2, 4] | 4 |
| $N_{l}$ | Number of Transformer layers | [1, 2] | 2 |
