# Supplementary material for: TPpred-LE: therapeutic peptide function prediction based on label embedding
Source: BMC Biol. 2023 Oct 31;21:238. doi: 10.1186/s12915-023-01740-w (PMC10617231; doi:10.1186/s12915-023-01740-w)
Supplement: Supplementary file 4 — Additional file 4: Fig S1. The distribution of different multi-functions and their relationship. Fig S2. The length distribution of the benchmark dataset. [file 12915_2023_1740_MOESM4_ESM.docx]

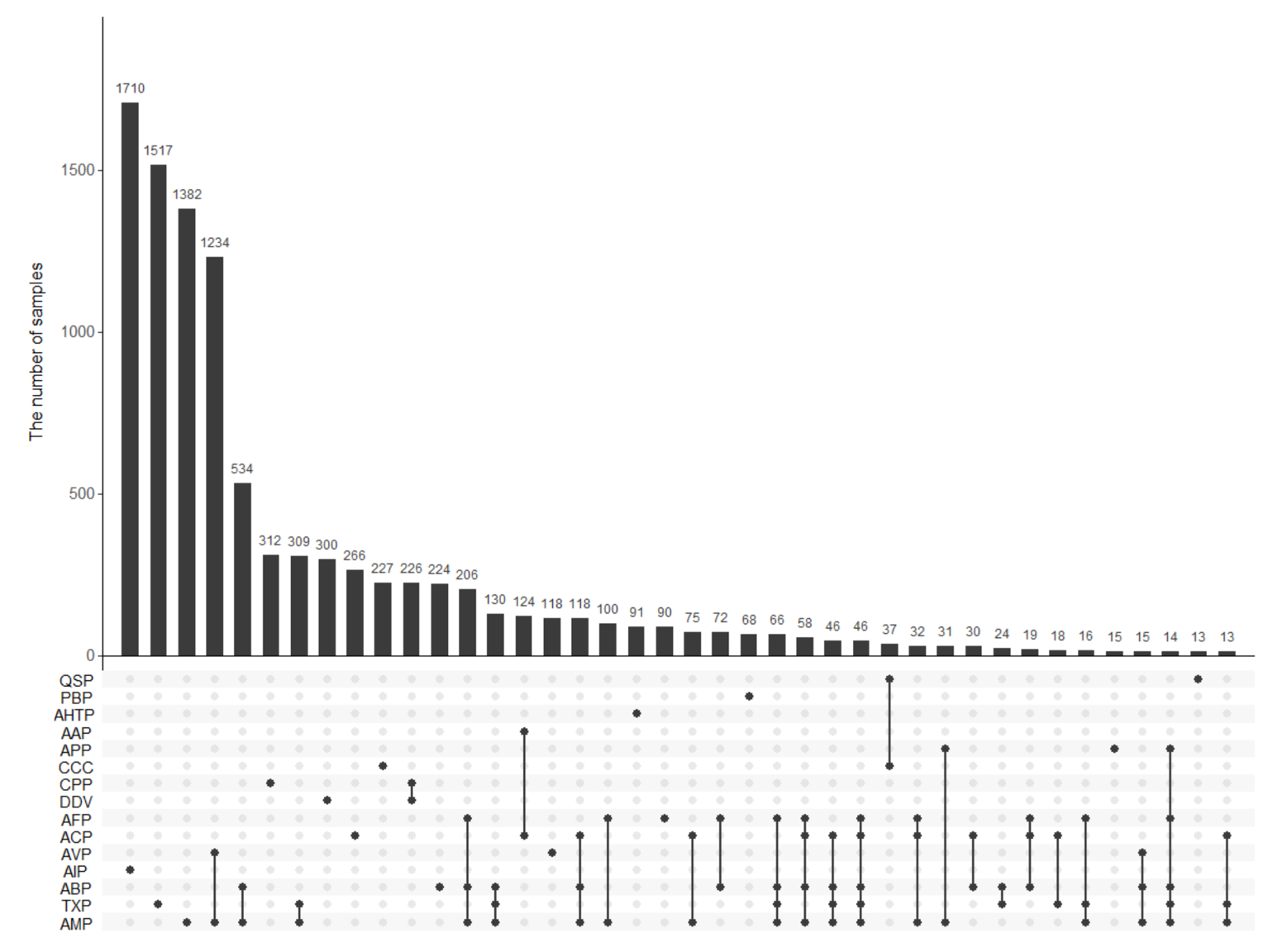


**Fig S1.** The distribution of different multi-functions and their relationship. There are two parts in this figure. The upper part shows the number of samples in each intersection subset. Only the intersection subsets with more than 12 samples are shown in this part for brevity. The bottom part shows detailed information on the 15 functions, where the black spots indicate that the corresponding functions listed in the y-axis share the intersection subset listed in the same column in the upper part. For examples, for column 4, there are two black spots in the bottom part, indicating that the functions AVP and AMP share the intersection subset with 1234 samples in the benchmark dataset.


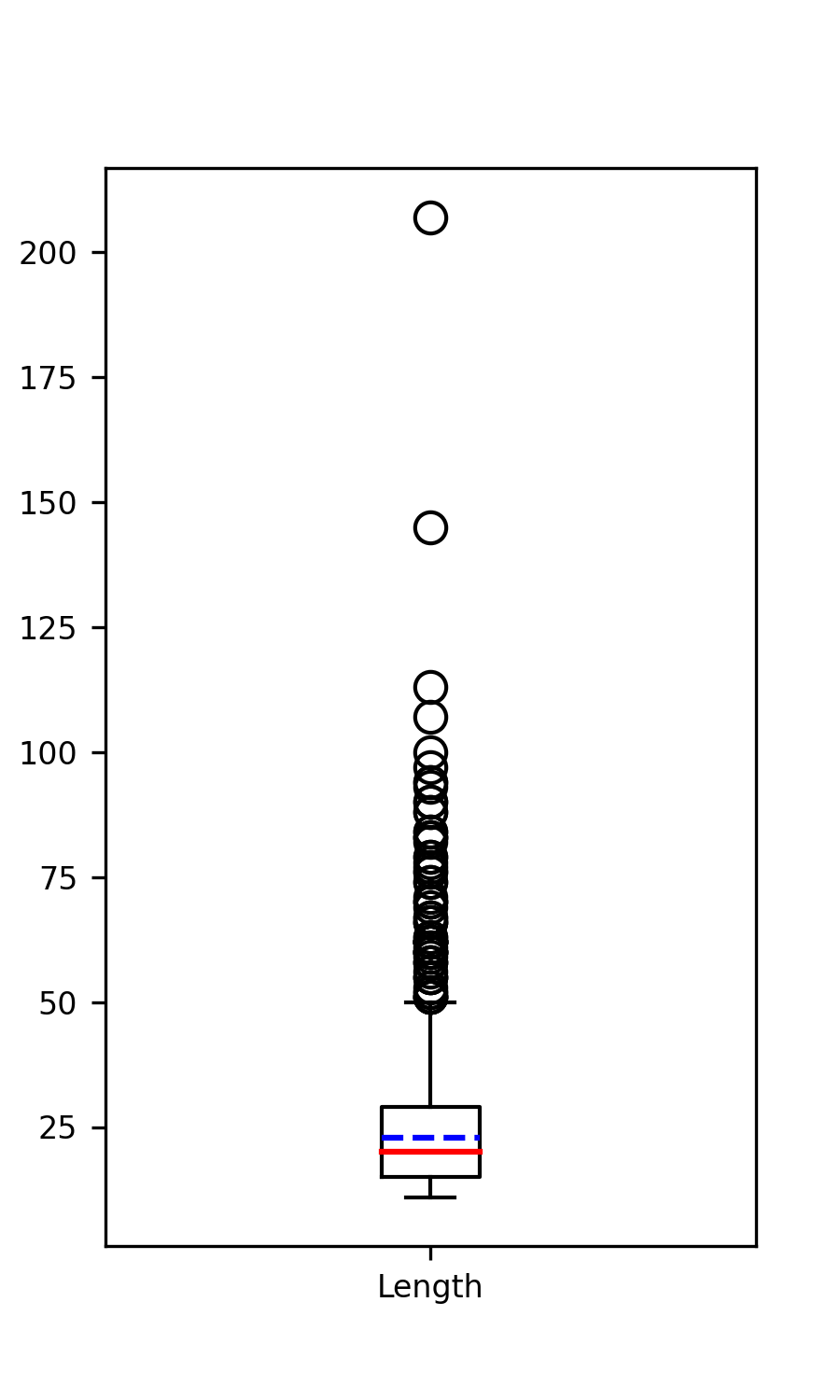


**Fig S2.** The length distribution of the benchmark dataset. The red line indicates the media length, and the blue line marks the average length. The vast majority of sequences have a length less than 50 in the benchmark dataset.
